# Pilot study to estimate the safety and effectiveness of hydroxyurea and methotrexate recurrent langerhans cell histiocytosis (LCH-HU-pilot)

**DOI:** 10.1097/MD.0000000000031475

**Published:** 2022-12-16

**Authors:** Kenichi Sakamoto, Kayoko Kikuchi, Mayumi Sako, Miho Kato, Tetsuya Takimoto, Yoko Shioda

**Affiliations:** a Children’s Cancer Center, National Center for Child Health and Development, Tokyo, Japan; b Department of Pediatrics, Shiga University of Medical Science, Otsu, Japan; c Department of Clinical Research Promotion, Clinical Research Center, National Center for Child Health and Development, Setagaya, Tokyo, Japan; d Department of Cancer Data Management, National Center for Child Health and Development, Tokyo, Japan.

**Keywords:** hydroxyurea, langerhans cell histiocytosis, methotrexate, relapse

## Abstract

Langerhans cell histiocytosis (LCH) is the most frequent type of histiocytosis and is characterized by both “inflammation/immune dysregulation” and “neoplastic disorder.” Although overall survival has remarkably improved through consecutive previous studies, the relapse-free survival rate is still only 60% to 70%, even in the latest clinical trials. Relapse of LCH is the most significant risk factor for permanent consequences in the central nervous system, such as diabetes insipidus, anterior pituitary hormone deficiency, and neurodegenerative diseases. In this trial, we evaluated the safety and effectiveness of hydroxyurea and methotrexate in recurrent LCH. Treatments in this study consisted of only orally administered drugs and not intravascularly administered drugs. Hydroxyurea therapy for patients with LCH is expected to be safer, less painful, and more cost-effective than other treatments for LCH. The results of this study could provide new therapeutic alternatives for recurrent LCH.

**Methods and analysis:**

This study was a non-blinded, multicenter, single-arm study. Recurrent (relapsed) LCH is defined as the appearance of new lesions or the enlargement of preexisting lesions due to LCH. In this study, all patients received hydroxyurea, and if the treatment response was unsatisfactory, methotrexate was added. The duration of treatment was 48 weeks. The primary endpoint was the rate of non-active disease achievement, which was 24 weeks after initiating hydroxyurea administration. No active disease is defined as the resolution of all the signs and symptoms related to LCH.

**Key Points**

Hydroxyurea (Hydrea^®^) has already been used for various diseases for decades, and a few serious adverse events caused by hydroxyurea (Hydrea^®^) have been reported.

Treatments in this study will consist of only orally administered drugs and not intravascularly administered drugs. Because some patients with LCH have multiple relapses, hydroxyurea and methotrexate therapy for LCH patients is expected to be safer, less painful, and more cost-effective than other treatments, including the intravenous administration of drugs for LCH.

The limitation of this trial is that it was a non-blinded, multicenter, single-arm study with a few samples size.

## 1. Introduction

Langerhans cell histiocytosis (LCH) is the most frequent type of histiocytosis, which is characterized by the accumulation of CD1a and CD207 positive histiocytosis with various inflammatory cells.^[1]^ LCH is derived from immature myeloid dendritic cells with an oncological mutation in the mitogen-activated protein kinase pathway, which induces sustained activation of the extracellular signal-regulated kinase. Additionally, the LCH lesion is accompanied with inflammatory cell infiltration and the release of several cytokines and chemokines.^[2]^ In this way, LCH shows the 2 aspects of “inflammation/immune dysregulation” and “neoplastic disorder.” LCH is now redefined as an “inflammatory myeloid neoplasia.”^[3]^

The prognosis for LCH has remarkably improved through consecutive clinical trials conducted by the Histiocyte Society (LCH-I,^[4]^ LCH-II,^[5]^ LCH-III^[6]^) using “vincristine/vinblastine based chemotherapy” and the Japan LCH Study Group (JLSG-96^[7]^ and JLSG-02^[8,9]^) using “cytarabine based chemotherapy.” Although the overall survival of LCH patients has improved to over 90%, the relapse-free survival rate remains at 60% to 70%, even in the latest clinical trials.^[6,8,9]^ The major relapse organs of LCH are the bone and skin, and relapse involving high-risk organs of LCH (liver, spleen, and hematopoietic system) is rare. In recurrent LCH, the treatment response is high and does not cause active disease in 85% of the cases.^[10]^ The most significant risk factor for permanent central nervous system consequences, such as central diabetes insipidus, anterior pituitary hormone deficiency, and neurodegenerative disease is a relapse of LCH.^[11,12]^

Zinn et al recently reported that hydroxyurea, which has previously been shown to be effective in the treatment of myeloid neoplasms (chronic myelogenous leukemia, polycythemia vera, and essential thrombocytosis) and sickle cell disease (SCD), is also effective in the treatment of LCH^.[13]^ In this report, fifteen recurrent or refractory LCH cases (13 adult cases and 2 childhood cases) involving the skin and bone with or without lymph node involvement were treated with hydroxyurea. After worsening signs and symptoms were observed due to LCH, 10 patients were treated only with hydroxyurea, and 5 patients were treated with combination therapy of hydroxyurea and methotrexate. The average duration of therapy was 10 months (range: 1–24 months). Twelve of the 15 (80%) patients achieved either complete or partial responses. After treatment with hydroxyurea, 6 (40%) patients showed progression or relapse after the initial response to hydroxyurea. This report demonstrates that treatment with hydroxyurea may be an effective therapeutic option for some LCH patients with relapse.

Therefore, we conducted a pilot study to evaluate the safety and effectiveness of hydroxyurea and methotrexate for recurrent LCH.

## 2. Methods

### 2.1. Purpose

In this study, we evaluated the safety and effectiveness of hydroxyurea and methotrexate in recurrent LCH. Recurrent (relapsed) LCH is defined as the appearance of new lesions or the enlargement of preexisting lesions due to LCH.

### 2.2. Study design

This was a non-blinded, multicenter, single-arm study. In this study, all patients received hydroxyurea, and if the treatment response was poor, methotrexate was added. We estimated the rate of non-active disease (NAD) at 24 weeks. NAD is defined as the resolution of all signs and symptoms related to LCH (refer to “Evaluation criteria”).

### 2.3. Endpoints

#### 2.3.1. Primary endpoint.

The rate of NAD achievement at 24 weeks after initiating hydroxyurea administration.

#### 2.3.2. Secondary endpoints.

1. Treatment response rate after 6 weeks of hydroxyurea treatment. The evaluation criteria of treatment response rate after 6 weeks are divided into good response (GR), partial response (PR), stable disease (SD), and progressing disease (PD), as detailed in “Evaluation criteria.”
2. Treatment response rates after 12, 24, 32, 40, and 48 weeks of hydroxyurea treatment. The evaluation criteria of treatment response rates at these time points are divided into NAD, active disease regression (AD-r), Active disease stability (AD-s), and active disease progression (AD-p), as detailed in “Evaluation criteria.”
3. The number of patients treated with methotrexate.
4. Frequency of adverse events associated with hydroxyurea administration.
5. The frequency of adverse events associated with the combination of hydroxyurea and methotrexate.
6. The number of hospital days and outpatient days during the study period.
7. Swimmer plot of all enrolled patients.
8. Relationship between hydroxyurea blood concentration and NAD, AD-r, AD-s, and AD-p achievement rates at 12, 24, and 48 weeks of treatment.

### 2.4. Eligibility criteria

We registered patients who satisfied all inclusion criteria and did not meet any of the exclusion criteria, as described below.

#### 2.4.1. Inclusion criteria.

1. The patient can take the capsule formulation.
2. Histopathologically diagnosed LCH at the time of initial or recurrent disease.
3. Recurrence of LCH (regardless of the number of recurrences).
4. Treatment for the recurrence of LCH has not been started, excluding local therapy.
5. Without the high-risk organ involvement of LCH, including the liver, spleen, and hematopoietic system.
6. Without the complication of hemophagocytic lymphohistiocytosis.
7. Fully maintained organ functions, including liver, kidney, cardiac, and respiratory functions.
8. Eastern Cooperative Oncology Group performance status (PS) score of 0 to 2.
9. Written informed consent to participate in this study is provided by the patient and/or legal representatives.

#### 2.4.2. Exclusion criteria.

1. Central diabetic insipidus recurrence on its own.
2. With active infectious disease.
3. With signs/symptoms of active bleeding.
4. With active signs/symptoms of the central nervous system.
5. With a history of hydroxyurea (Hydrea^®^).
6. Patients otherwise deemed unsuitable for this study by the investigators.

### 2.5. Sample size

The planned number of subjects for registration was 10 patients.

### 2.6. Registration of patients

The investigator obtained written informed consent from patients who fulfilled the inclusion criteria or their legally acceptable representatives before registering them in this study.

### 2.7. Treatment method

**Figure 1.**
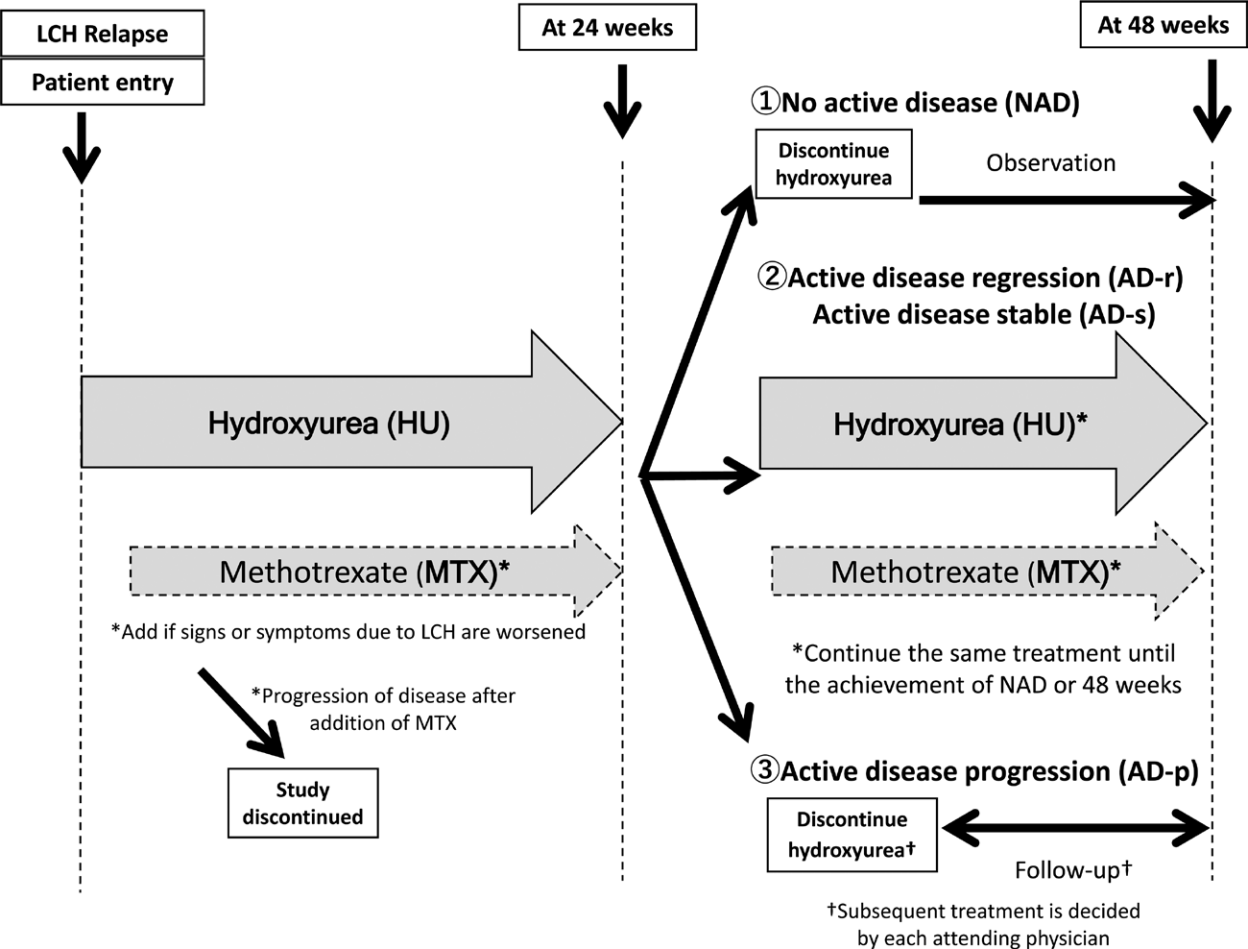
The schema of this study.

Schematics of this study are presented in Figure 1. The effect of Hydroxyurea was administered for 24 weeks. The dose of hydroxyurea for adults was a fixed-dosage regimen of 1000 mg per dose (twice a day). Because hydroxyurea is only available as a capsule formulation in Japan, the dose of hydroxyurea for pediatric patients (<18 years) was modified according to body weight: If the body weight of the patient is > 40 kg, 20 to 40 kg, or < 20 kg, the patient will receive 1000 mg per dose (twice a day), 500 mg per dose (once a day), or 500 mg per dose (3 times a week), respectively. If the signs and symptoms due to LCH worsened after starting hydroxyurea, we started a combination therapy of hydroxyurea with methotrexate. After 24 weeks of treatment initiation, we evaluated the treatment response in all patients (refer to evaluation criteria). Patients who achieved NAD at 24 weeks were made to discontinue treatment with hydroxyurea and methotrexate and were observed until 48 weeks. If the patients achieved AD-r or AD-s at 24 weeks, we continued the same treatment until the achievement of NAD or 48 weeks. Patients who developed AD-p were also made to discontinue treatment with hydroxyurea and methotrexate and were observed until 48 weeks. For these patients, an alternative treatment regimen was decided by each attending physician. The duration of treatment in this study was 48 weeks. At 48 weeks, the treatment response was reevaluated and information was collected on adverse events during hydroxyurea and methotrexate treatment. Prohibit the administration of the drugs for LCH other than this protocol study drugs, such as hydroxyurea and methotrexate.

### 2.8. Evaluation criteria

#### 2.8.1. Initial treatment response at six weeks after hydroxyurea administration.

Treatment response at 6 weeks after hydroxyurea administration is evaluated as follows:

i) Good response (GR)

GR was defined as the disappearance of the signs or symptoms of LCH. Bone lesions on radiology were not clear at the 6-weeks evaluation after hydroxyurea administration. Therefore, the disappearance of bone lesions in radiology is not required.

ii) Partial response (PR)

PR was defined as patients meeting all the following criteria: ≥30% decrease in the sum of the longest diameter of the LCH lesions, regression (>50%) of skin or mucosa LCH lesions, and no new lesion of LCH compared with baseline. Bone lesions on radiology were not reduced at the 6-weeks evaluation after hydroxyurea administration. Therefore, reduction of bone lesions in radiology is not required at this point as well.

iii) No response (NR)

Patients with NR satisfy the following criteria: ≤30% decrease in the sum of the longest diameter of the LCH lesions or regression (<50%) of skin or mucosa LCH lesions with or without the appearance of any new LCH lesion compared to baseline.

iv) Progressing disease (PD)

PD is defined as the worsening of signs or symptoms due to LCH and/or the appearance of new LCH lesions.

#### 2.8.2. Treatment response at 12, 24, 32, 40, and 48 weeks after hydroxyurea administration.

Treatment response at these time points after hydroxyurea administration is evaluated as follows.

i) No active disease (NAD)

NAD is defined as the complete resolution of signs and symptoms due to LCH.

ii) Active disease regression (AD-r)

AD-r is defined as a continuous regression of signs and symptoms due to LCH, except for permanent consequences.

iii) Active disease stable (AD-s)

AD-s is defined as LCH-related signs and symptoms that remain stable in the absence of new LCH lesions.

iv) Active disease progression (AD-p)

AD-p is defined as the worsening of signs or symptoms caused by LCH or the development of any new LCH lesion.

In addition, relapse is characterized as the appearance of new LCH lesions (including central diabetes insipidus) or enlargement of baseline LCH lesions after the achievement of GR/PR or NAD.

### 2.9. Adverse event reported

The investigators collected all adverse events that had occurred in the patients during the study period. Adverse event severity was determined using the Common Terminology Criteria for Adverse Events (CTCAE) version 5.0.

### 2.10. Statistical analysis

We analyzed the rate of NAD achievement 24 weeks after initiating hydroxyurea administration, which is the primary endpoint when the data of all registered patients up to 48 weeks after starting hydroxyurea administration are fixed. Analysis of secondary endpoints was performed after the final follow-up data for all patients was fixed.

### 2.11. Data collection

Data collected and stored via REDcap software during this study. REDCap has been privacy and security protected. The datasets generated during and/or analyzed during the current study are not publicly available, but are available from the corresponding author on reasonable request.

### 2.12. Monitoring

This study monitored on a regular basis. Monitoring ensured that the study was conducted safely and in accordance with the protocol. It also determined whether the data are accurately recorded and properly stored. Any changes to the protocol would be reapproved through the Central Institutional Review Board of the National Center for Child Health and Development.

### 2.13. Patients or the public involvement

The patients were not involved in the design, conduct, reporting, or dissemination plans of our research.

### 2.14. Ethics and dissemination

This trial was performed in accordance with the ethical principles of the Declaration of Helsinki and the Clinical Trial Act. This trial was approved by the Central Institutional Review Board of the National Center for Child Health and Development. Informed consent was obtained from the patients and/or their parents before registration. All data was collected confidentially from the investigators using an electronic data capture system. We will present the results of this trial at national and international conferences and publish them in an international, peer-reviewed journal.

## 3. Discussion

We conducted a pilot study to evaluate the safety and efficacy of hydroxyurea and methotrexate in recurrent LCH. Various treatments for LCH patients with relapse have been reported, such as using the same agents from the first-line therapy (prednisolone, vinblastine, methotrexate, and 6-mercatopurine),^[10]^ bisphosphonate,^[14]^ indomethacin,^[15]^ 2-chlorodeoxyadenosine,^[16]^ and clofarabine.^[17]^ However, there has been no clear standard therapy until now. Hydroxyurea, a myelotoxic ribonucleotide reductase inhibitor, was first approved in 1966. Daily oral hydroxyurea treatment has a low toxicity profile. Similar to adults, treatment with hydroxyurea is safe and tolerable in children. The Hydroxyurea Safety and Organ Toxicity (HUSOFT) trial revealed that the toxicity profile of long-term hydroxyurea therapy (mean duration of hydroxyurea was 4.9 ± 1.3 years) for infants with SCD was limited, and that long-term hydroxyurea usage did not affect their growth rate.^[18]^ Additionally, other reports showed that pubertal development in patients with SCD was also maintained after long-term use of hydroxyurea.^[19,20]^

Moreover, the treatments in this study consisted of only orally administered drugs and not intravascularly administered drugs. Some patients with LCH have multiple relapses and require frequent hospital visits and hospitalization to receive intravenous drugs. From this perspective, the burden on patients is quite significant, and less burdensome treatment is desired. In this study, the burden on patients was considerably minimized by using an oral formulation of the drug. Regarding cost, one capsule of hydroxyurea (500 mg per capsule) costs 241 Japanese yen, making the treatment highly cost-effective.

Based on these findings, hydroxyurea therapy for LCH patients is expected to be safer, less painful, and more cost-effective than other treatments, including intravenous administration of drugs for LCH. The results of this study could provide new therapeutic alternatives for recurrent LCH.

## Authors contributions

K.S., Y.S., K.K., and M.S. conducted the pilot study. K. S. and Y. S. managed the study. T.T. and M.K. developed an electronic data-capture system for this study.

Conceptualization: Kenichi Sakamoto, Yoko Shioda.

Data curation: Miho Kato, Tetsuya Takimoto.

Funding acquisition: Yoko Shioda.

Project administration: Kenichi Sakamoto, Kayoko Kikuchi, Mayumi Sako, Yoko Shioda.

Writing – original draft: Kenichi Sakamoto.

Writing – review & editing: Kayoko Kikuchi, Mayumi Sako, Miho Kato, Tetsuya Takimoto, Yoko Shioda.
